# Evaluation of Marginal Leakage and Shear Bond Strength of Bonded Restorations in Primary Teeth after Caries Removal by Conventional and Chemomechanical Techniques

**DOI:** 10.1155/2014/854816

**Published:** 2014-08-07

**Authors:** Viral Pravin Maru, Bethur Siddaiah Shakuntala, Nagarathna Dharma

**Affiliations:** Department of Pedodontics and Preventive Dentistry, Rajarajeswari Dental College and Hospital, Mysore Road, Kumbalgodu, Bangalore, Karnataka 560074, India

## Abstract

*Background/Purpose*. To evaluate and compare the marginal leakage and shear bond strength between conventional and Papacarie techniques of caries removal in primary molars. *Materials and Methods*. Sixty freshly extracted human carious primary molars were randomly divided into two groups: group I—caries removal by conventional method and group II—caries removal using Papacarie. After bonded restorations, both groups were further randomly subdivided into four subgroups for marginal leakage and shear bond strength evaluation. *Results*. Papacarie treated teeth (46.70%) showed less marginal leakage when compared to conventionally treated teeth (86.70%) for caries removal. The mean shear bond strength was found more in Papacarie treated teeth (12.91 MPa) than in those treated conventionally (9.64 MPa) for caries removal. *Conclusion*. Papacarie showed less marginal leakage and more shear bond strength when compared to those treated conventionally for caries removal.

## 1. Introduction

Caries excavation has traditionally been performed according to mechanical principles using drills and sharp-edged hand instruments. These methods, although often effective, have some major disadvantages. First, it is often difficult to establish the amount of dentin to be removed due to the apparent lack of objective clinical markers. Secondly, local anaesthesia is needed to alleviate the pain and discomfort caused by mechanical methods [1]. In order to circumvent these drawbacks, alternative dental caries removal methods, such as chemomechanical techniques, air/sono abrasion, ultrasonic instrumentation, and lasers have been proposed [2]. Chemomechanical elimination of carious dentin has by far been the most promising alternative treatment procedure, particularly in paediatric dentistry and for anxious or medically compromised patients [3].

Chemomechanical method of caries removal by using 5% sodium hypochlorite was first introduced in 1975 by Habib et al. [4]. This was followed by the introduction of GK-101, Caridex system, and Carisolv, consisting of sodium hypochlorite, glutamic acid, leucine, and lysine [4–7]. In consequence of certain disadvantages like short shelf life, high corrosiveness, requirement of specialized instruments, and high cost, a research project in Brazil by Bittencourt et al. led to the development of a new formula, commercially known as Papacarie. This chemomechanical method for caries removal contains 10% papain, 0.5% chloramine-T, toluidine blue, and a thickening agent [8, 9]. A number of studies have compared the efficacy of chemomechanical methods with conventional rotary technique and highlighted the merits of the former with respect to reduced pain and need of anaesthesia and patient acceptance [10–13].

Bonded restoration after removal of caries is largely determined by the strength of adhesion between bonding material and the surface of tooth substrate. Lack of interaction between adhesive system and tooth substrate yields poor marginal sealing leading to marginal leakage, which, in turn, may result in early loss of the restoration, postoperative sensitivity, discoloration, marginal deterioration and secondary caries, and ultimately to displacement of the restoration and pulpal damage. After caries removal using Papacarie, residual dentine surface did not possess a smear layer and patent tubules, whereas smear layer was partially or fully present on treatment with Carisolv [14]. On the other hand, use of conventional rotary drill is a harsh procedure without much control and is known to cause damage to the surface. In scanning electron microscopy studies, rotary drilling has resulted in smooth and regular dentine surface with a smear layer and is expected to form weaker bonding with adhesive systems [15]. In addition, the effectiveness of binding of adhesive material after the use of Papacarie is not fully characterized [15]. Thus, micromorphological alterations caused by the use of chemomechanical agents are expected to influence marginal leakage and shear bond strength characteristics and, in turn, the quality of restorations. The present study has been designed to test this hypothesis by evaluation and comparison of the marginal leakage and shear bond strength in primary molar bonded restorations after caries removal by conventional as well as Papacarie methods.

## 2. Materials and Methods

The present study was carried out in the Department of Pedodontics and Preventive Dentistry, Rajarajeswari Dental College and Hospital, Bangalore, India, with the approval of the ethics committee of the same institution. Sixty freshly extracted, human primary molars with occlusal caries extending into the dentin, with cavity openings diameter ≥2 mm, and with accessibility to hand instruments were collected. These teeth were extracted due to exfoliative mobility and/or orthodontic reasons. Paediatric patients were selected by random sampling technique. An informed consent was taken from the patient's parent/guardian prior to the extraction procedure. The primary molars with occlusal caries extending into dentin were confirmed through intraoral periapical radiograph whereas for teeth involving pulpal and/or periapical pathology, multisurface carious lesions and teeth with developmental anomalies were excluded. The surfaces of teeth were cleaned with Hu-Friedy universal scaler number 11 blade for removal of calculus and remnants of periodontal ligament. These teeth were stored in 2% formalin (pH = 7.0) for 14 days and subsequently in saline solution. Sixty primary molars were then randomly divided into two experimental study groups. Group I consisted of 30 teeth for which carious tissue was removed by conventional method, that is, using a high speed hand piece under cooling system with a number 330 carbide bur. The cavity was rinsed with water and wiped with sterile cotton pellet. Group II consisted of 30 teeth for which the carious tissue was removed using the chemomechanical Papacarie technique. Papacarie (Formula and Acao) gel was dispensed onto a dappen dish. It was then applied onto the dentinal carious lesion using a plastic filling instrument. The lesion was completely covered by the gel for thirty seconds. When the gel was cloudy, it was removed gently by scrapping with the spoon excavator without applying pressure. The softened tissue was scrapped, but not cut. The gel was reapplied for another thirty seconds till the cavity appeared vitreous which indicated that the cavity was completely free of caries. The cavity was rinsed and wiped with sterile cotton pellet [9]. The completeness of removal of caries was judged by visual (absence of any discoloration) and tactile (smooth passage of the explorer and absence of a catch or a tug-back sensation) methods in both groups [7].

Adper easy one self-etch adhesive (3M ESPE) was applied to all surfaces of the cavity with a disposable applicator for 20 seconds. The disposable applicator was rewet as needed during application. Care was taken to avoid contact of the adhesive with mucosal tissue. Subsequently, the liquid was air-thinned for 5 seconds until the film no longer moved, indicating complete vaporization of the solvent. The adhesive was cured for 10 seconds. The cavities were then restored with composite Z250 (3M ESPE), as per the manufacturer's instructions. After restorations, both experimental groups were stored in saline at 37°C for 72 hours separately. Later they were polished with abrasive rubber cup in slow speed hand piece in order to remove the saline remnants. Both groups were subjected to thermocycling in distilled water at 5 and 55°C (±2°C), for 100 cycles for 30 seconds each. The two groups were further subdivided randomly into the following subgroups: (i) groups IA and IIA (15 teeth each) for marginal leakage test and (ii) groups IB and IIB (15 teeth each) for shear bond strength test.

### 2.1. Marginal Leakage Test

Experimental group IA and group IIA received two coats of nail varnish on the entire tooth surface except for the restoration and a 1 mm rim of tooth structure around the restoration and was allowed to air-dry. The apices up to the furcation were sealed with sticky wax. Teeth from both groups were then immersed in 2% basic fuchsine dye for 8 hours separately. After 8 hours, teeth were washed in tap water for 10 minutes and air-dried. This was followed by longitudinal sectioning of teeth in two sections at the centre of the restoration with diamond disc in slow speed hand piece and water coolant. Stereomicroscope (40x) was used to evaluate the amount of marginal leakage. Scores based on a scale [15] from 0 to 3 were assigned depending on the amount of dye penetration: 0: no penetration, 1: penetration into the surrounding enamel, 2: penetration into the dentin, and 4: penetration into the cavity floor. Both sections were scored, and the worst score was recorded.

### 2.2. Shear Bond Strength Test

Extracted and restored teeth from group IB and group IIB were stored in distilled water for 2 days after thermocycling. The teeth were then mounted on acrylic resin blocks and subjected to shear bond strength test using Lloyd testing machine (LR50K) with a crosshead speed of 1 mm/min. The specimen mounted on its acrylic block was secured to the lower grip of the machine. The force required to debond was recorded.

### 2.3. Statistical Analyses

Descriptive statistical analysis was performed on marginal leakage and bond strength data from all the four subgroups. Chi square test and Fischer's exact test were used to assess marginal leakage and unpaired *t*-test was used for assessing shear bond strength at 0.05 level of significance. All statistical analyses were performed using SAS software system.

## 3. Results

**Figure 1:**
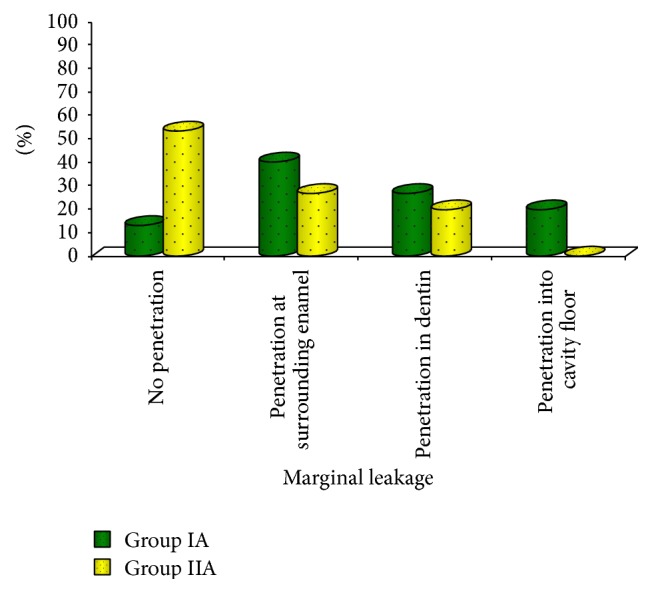
Comparison of marginal leakage between groups IA and IIA.

**Figure 2:**
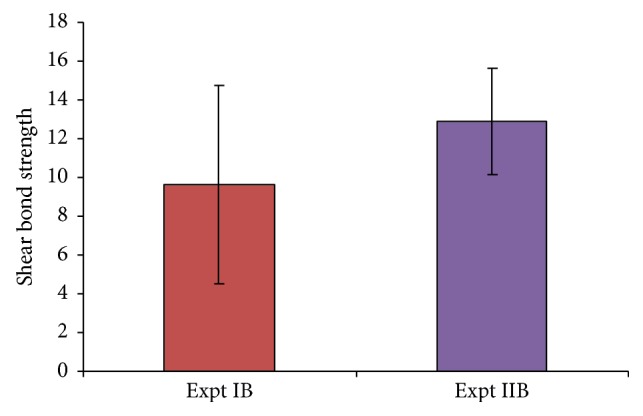
Comparison of mean shear bond strength between groups IB and IIB.

Figure 1 shows that Papacarie treated teeth had less marginal leakage (46.70%) compared to conventionally treated teeth (86.70%) although the difference is not statistically significant (*P* > 0.05). Teeth treated with Papacarie as well as conventional methods showed more marginal leakage at the surrounding enamel when compared to those at the dentin and at the base of the cavity floor. Papacarie treated teeth had high shear bond strength (12.91 ± 2.75 MPa) when compared to conventionally treated teeth (9.64 ± 5.13 MPa) as shown in Figure 2.

## 4. Discussion

In the present study, higher marginal leakage was observed with conventionally treated teeth than those treated with Papacarie. Papacarie removes smear layer, in contrast to conventional method of caries removal wherein smear layer is produced that affects the polymerisation of the bonding mechanism. The highly irregular surfaces or high roughness maintained in the absence of a smear layer in Papacarie treated cavities could provide a suitable surface for good adhesion in strong bonding with restorative materials; hence, less marginal leakage was observed [4].

In order to conduct the investigation under the conditions of daily clinical practice, the completeness of caries removal was judged by standard clinical criteria. It has been suggested that conventional visual and tactile criteria are sufficient to ensure the removal of most infected dentin [16]. Dyes were not used, as their use does not provide a complete objective method for assessment of caries removal. The dye penetrates deeply and stains carious infected dentin as well as the porous affected dentin. Primary dentin being porous, use of dye would not be suitable for assessment of complete removal [17]. At the same time, the extracted teeth may respond to caries excavation differently than the teeth in function, since an outward flow of fluid has been reported in* in vivo* dentin, which is partly ameliorated by using freshly extracted teeth [18].

Papacarie acts by breaking the partially degraded collagen molecules, contributing to the degradation and elimination of the fibrin “mantle” formed by the carious process. The attack causes cleavage of the polypeptide chains and hydrolyses the crosslinks of collagen fibrils. After the degradation, oxygen is freed, and this explains the appearance of bubbles on the surface and blearing of the gel during the clinical procedure. These signs demonstrated that the removal process has been started. The chemical agent was found to have no ability to affect the sound collagen fibres in the inner affected and normal dentin, as papain can digest only dead cells because infected tissues lack or do not show antitrypsin which inhibits protein digestion [8].

Self-etch adhesive system does not completely resolve or remove the smear layer, but rather partly integrates into the hybrid layer and it has relatively high bond strength to enamel and dentin and has been designed to simplify clinical procedures and hence used in this study [19]. Self-etching system lacks the rinsing step and thus the smear layer is not removed due to which high amount of marginal leakage was reported in group subjected to conventional method of caries removal.

The shear bond strength (mean ± SD) was significantly more in group IIB (12.91 ± 2.75 Mpa) when compared to Group IB (9.64 ± 5.13 MPa). This result was in accordance with the study conducted by Lopes et al. in 2007, who reported shear bond strength of 10.87 ± 5.97 MPa between Papacarie treated demineralized slabs and resin composite [20]. Bond strength values depend on laboratory equipment and instrumentation, reflecting specimen geometry, sample preparation, surface area, storage protocols, strain used to debond specimens, and operator variability [21]. This study used thermocycling to mimic the 24-hour intraoral environment. The specimens were thermocycled 100 times, since more than 100 cycles have been shown to be unnecessary [22].

The use of natural lesions in the present study did not allow standardization of all the variables of sample, for example, shape of lesions, activity status of the lesions, location, type of lesions, consistency, and depth. Hence, long-term clinical studies are required to critically evaluate the relevance of these* in vitro* results.

## 5. Conclusion

In conclusion, present observations confirm that caries removal methods and the features of residual surface after the treatment have a distinct influence on the binding characteristics of adhesion systems. Papacarie treated restorations showed less marginal leakage when compared to conventionally treated teeth in bonded restorations. Shear bond strength of Papacarie treated teeth was higher than that of conventionally treated teeth on bonded restoration.
